# Understanding Bone Density Loss in Eating Disorders: Contributions of Weight Suppression and Speed of Weight Loss

**DOI:** 10.3390/jcm13247537

**Published:** 2024-12-11

**Authors:** Patrizia Todisco, Paolo Meneguzzo

**Affiliations:** 1 Eating Disorder Unit, Casa di Cura “Villa Margherita”—KOS Group, 36057 Arcugnano, Italy; patrizia.todisco1964@gmail.com; 2 Department of Neuroscience, University of Padova, 35122 Padova, Italy; 3 Padova Neuroscience Center, University of Padova, 35122 Padova, Italy

**Keywords:** eating disorders, bone density, vitamin D, weight suppression, weight loss speed, anorexia nervosa: bulimia nervosa, binge eating disorder

## Abstract

**Background/Objectives:** Eating disorders (EDs), including anorexia nervosa (AN), bulimia nervosa (BN), and binge eating disorder (BED), are associated with bone density loss. Weight suppression (WS) and weight loss speed (WLS) are two critical weight-related factors that may influence bone health, yet their relationship with bone density remains underexplored. This study aimed to investigate the associations between WS, WLS, and bone density in individuals with EDs, focusing on total body and spinal bone density. **Methods:** We examined 270 individuals with EDs (AN: n = 187, BN: n = 57, BED: n = 26) at the onset of inpatient treatment. WS and WLS were calculated from weight history, and bone density was assessed using dual-energy X-ray absorptiometry (DXA). Regression analyses were performed separately for each diagnosis. **Results:** In AN, both WS and WLS were significant predictors of total (*p* = 0.001) and spinal (*p* = 0.007) bone density. WS and WLS independently predicted total bone density, with WS significantly predicting spinal bone density. In BN and BED, only WLS showed significant associations with bone density. Minimum weight was a key predictor of bone density in AN, underscoring the importance of avoiding extremely low body weight. **Conclusions:** WS and WLS significantly affect bone density in AN, with WLS also predicting bone density in BN and BED. These findings highlight the need to monitor weight-related factors across ED populations. In AN, avoiding extremely low body weight is crucial for preserving bone health, while in BN and BED, managing WLS is key to mitigating bone density loss.

## 1. Introduction

Eating disorders (EDs) encompass a range of severe psychiatric conditions, including anorexia nervosa, bulimia nervosa, and binge-eating disorder, all of which profoundly impact both mental and physical health, with a reduction in the perceived quality of life [1,2]. The dual psychological and physical components of EDs are well-documented, with psychological factors such as distorted body image, emotional dysregulation, internal voices, and perfectionism interplaying with physiological consequences like malnutrition, hormonal dysregulation, and systemic damage [3,4,5]. Sociocultural factors, including societal beauty standards and media portrayals, further exacerbate these disorders by shaping unrealistic body ideals and promoting disordered eating behaviors, as supported by recent meta-syntheses of eating disorder research [6,7,8,9]. While much research has focused on the psychological and behavioral manifestations of EDs, an equally critical aspect is the physical health of individuals affected by these disorders [10,11,12]. Among the numerous medical complications, bone health deterioration stands out as particularly concerning [13]. Bone density loss, prevalent among ED patients, often leads to osteoporosis, fractures, and chronic pain, with significant long-term implications [14]. The risk of bone fragility and associated disabilities not only affects the quality of life but can also complicate the recovery process, highlighting the need to understand factors that may accelerate bone density loss [2,15].

Existing research underscores the importance of weight-related factors in bone health among individuals with EDs. For instance, one study found that normalized present and past minimum weight are the strongest predictors of lumbar bone density in individuals with EDs, even after controlling for age and height [16]. Other factors, such as caloric intake, binge eating, vomiting, menstrual status, and estrogen use, appeared to have no significant independent effect on bone density. These findings emphasize the critical role of weight history as a determinant of bone health and highlight the potential for weight suppression (WS) and weight loss speed (WLS) to illuminate these relationships further [17].

Weight suppression is defined as the difference between an individual’s highest historical weight and their current weight. In ED populations, high WS often indicates the extent of the body’s deviation from its biologically set weight, imposing physiological stress as the body adapts to sustain a lower weight. Existing studies have shown that individuals with greater WS often experience more severe psychopathology, such as increased obsession with weight, distorted body image, and extreme dietary restriction, all of which can intensify the physical strain associated with malnutrition and exacerbate medical complications [18,19,20].

In addition to WS, weight loss speed (WLS) is thought to play a critical role in the medical complications of EDs, as rapid weight reduction imposes acute physiological stress [17,19,21]. Accelerated weight loss may disrupt nutrient availability, impair gastrointestinal and endocrine functioning, and intensify bone resorption—an effect wherein bone tissue is broken down faster than it can be rebuilt [22,23]. Studies suggest that rapid weight loss can trigger hormonal imbalances, reduce calcium absorption, and disturb the balance of bone-forming cells (osteoblasts) and bone-resorbing cells (osteoclasts) [24,25]. This accelerated bone loss, when combined with malnutrition and low body weight, may increase the risk of osteoporosis and fractures among individuals with EDs [13,26].

Although previous studies have linked both WS and WLS to heightened clinical severity in EDs, much of the research has focused on psychological rather than physical outcomes [13,17,27,28]. This leaves a significant gap in understanding how these weight-related variables directly impact bone density and bone health. Recognizing the implications of WS and WLS on bone density is particularly important for patients with EDs, as bone density loss is often irreversible and can impose lifelong consequences, regardless of psychological recovery [29,30,31].

Therefore, this study aims to evaluate the relationship between WS, WLS, and bone density in individuals diagnosed with EDs. We hypothesize that higher WS and faster WLS are associated with lower bone density, due to the compounded effects of physiological stress, hormonal disruption, and nutrient deficiencies. The findings of this study will contribute to a more comprehensive understanding of how weight-related variables affect bone health in EDs, potentially informing interventions to protect bone density in vulnerable individuals. This research is essential for developing integrated treatment approaches that address both the psychological and medical needs of individuals with eating disorders.

## 2. Materials and Methods

### 2.1. Participants

The study involved 270 individuals diagnosed with eating disorders at the onset of their inpatient treatment at the Eating Disorder Unit of Casa di Cura Villa Margherita-KOS (Arcugnano-Vicenza) between 2014 and 2024. Participants were enrolled consecutively based on their admission to the rehabilitation program, and the sample size was determined by the availability of individuals within the specialized ED rehabilitation program over the past 10 years. Diagnoses were established by experienced eating disorder clinicians using a semi-structured clinical interview based on DSM-5 criteria, in line with standard clinical practice [32]. Of the participants, 187 were diagnosed with anorexia nervosa (AN), 57 with bulimia nervosa (BN), and 26 with binge-eating disorder (BED). The high proportion of AN participants reflects the recruitment setting within an inpatient clinic.

Exclusion criteria included the presence of severe acute psychiatric comorbidities, such as psychosis or mania, which would interfere with the execution of bone densitometry. All participants identified as cisgender, with the majority identifying as white (95.9%).

Participants were enrolled consecutively; however, due to local health regulations, DXA scans could only be performed on individuals residing in the Veneto region.

Written informed consent was obtained from all participants.

### 2.2. Measures

The clinical staff recorded patients’ weight and height measurements, as well as weight history data, including each patient’s highest and lowest weights at their current height. They also documented the duration between the highest and lowest weights. Weight suppression (WS) was calculated as the difference between the maximum and current weights, expressed as BMI to account for height. Weight loss speed (WLS) was calculated as the ratio of WS (in terms of BMI) to the total time (in months) over which the weight loss occurred. For both WS and WLS, only the most recent episodes of weight loss were considered, particularly in cases involving multiple episodes or prolonged illness duration [17].

All participants underwent a total body dual-energy X-ray absorptiometry (DXA) scan using a Hologic Horizon system to assess bone density. This non-invasive procedure uses low-dose X-rays to measure bone mineral density and composition, with data collected for total body and spinal column bone density. Bone density measurements included Z-scores, which indicate how a participant’s bone density compares to the average bone density of individuals of the same age and sex. The Z-score is calculated by expressing the difference between a participant’s bone density and the age- and sex-matched mean in standard deviation units, providing a standardized measure to assess deviations from typical bone density levels. Z-scores were used in this study because all participants were below the age of 50, as recommended by clinical guidelines for evaluating bone density in younger populations. Additionally, blood samples were taken to assess vitamin D concentrations, providing insight into participants’ nutritional status related to bone health.

### 2.3. Statistical Plan

Normality was assessed using the Shapiro–Wilk test, which indicated that the majority of the variables were non-normally distributed. Consequently, we performed a Kruskal–Wallis test to assess differences in key variables between individuals with AN, BN, and BED. Effect sizes were calculated using eta squared (η^2^), used to indicate small effects (0.01), medium effects (0.06), and large effects (0.14). Post hoc comparisons were performed to identify specific group differences when significant results were obtained with Bonferroni correction. Spearman’s rho was employed to examine the associations between bone density (total and spinal Z-scores) and other continuous variables, including WS, WLS, vitamin D levels, BMI, age, age of onset, maximum weight, minimum weight, and age of first fasting, within each diagnostic group. Multiple regression analyses were conducted to identify predictors of bone density outcomes. Separate regression models were performed for total and spinal bone density Z-scores, incorporating WS and WLS as independent variables. Additional regression analyses included age, BMI, age of onset, maximum weight, and minimum weight to explore the combined influence of these factors on bone density. Statistical significance was determined at an alpha level of *p* < 0.05 for all analyses, executed using IBM SPSS Statistics 25.0 (SPSS, Chicago, IL, USA).

## 3. Results

### 3.1. Descriptive Analyses

Descriptive statistics, including means, standard deviations, and ranges, were calculated for all key variables, including WS, WLS, vitamin D levels, age, BMI, age of onset, maximum and minimum weight, and age of first starvation. Table 1 presents the summary statistics for these primary study variables.

Examination of the distribution of Z-scores for total body bone density and spinal bone density revealed the following ranges for each diagnostic group: individuals with anorexia nervosa, the total bone density Z-scores ranged from −6.00 to 3.10 and spinal bone density Z-scores ranged from −5.30 to 2.00; individuals with bulimia nervosa, the total bone density Z-scores ranged from −2.80 to 2.60 and spinal bone density Z-scores ranged from −3.50 to 2.40; individuals with binge eating disorder, total bone density Z-scores ranged from −2.20 to 2.70 and spinal bone density Z-scores ranged from −1.90 to 2.90.

### 3.2. Correlation Analyses

**Figure 1:**
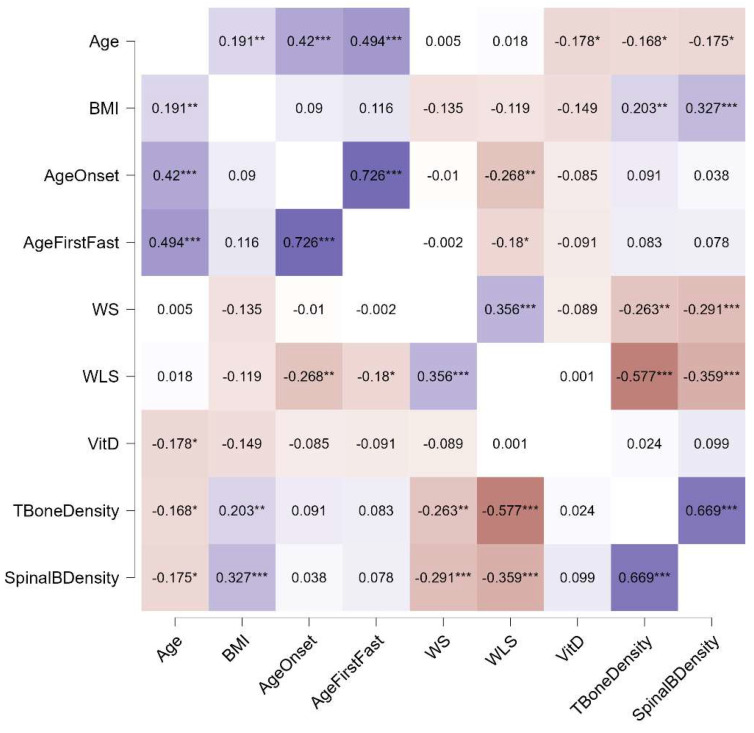
Spearman’s rho correlations for participants with AN. Purple indicates positive correlations, red indicates negative correlations, and the intensity of the colors reflects the strength of the correlation. WS, weight suppression; WLS, weight loss speed; VitD, vitamin D; *, *p* < 0.05; **, *p* < 0.01; ***, *p* < 0.001.

**Figure 2:**
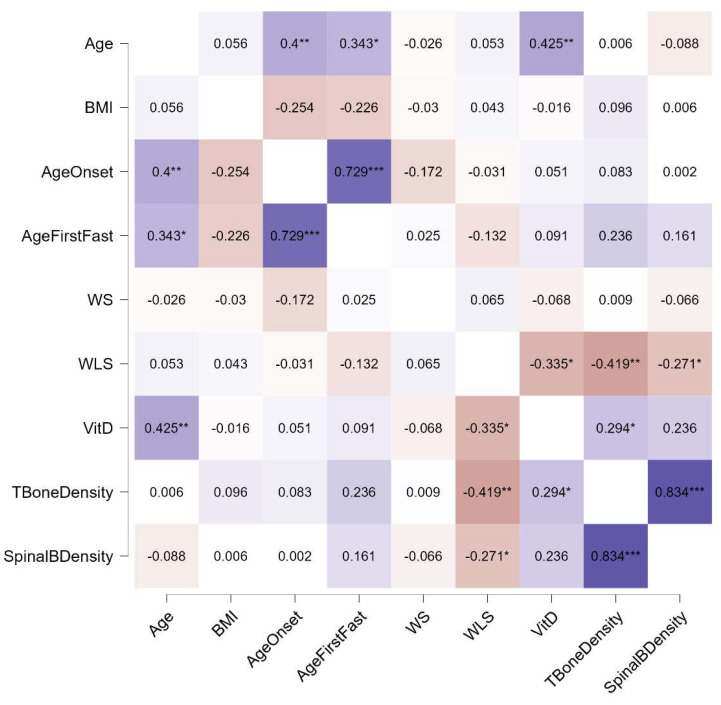
Spearman’s rho correlations for participants with BN. Purple indicates positive correlations, red indicates negative correlations, and the intensity of the colors reflects the strength of the correlation. WS, weight suppression; WLS, weight loss speed; VitD, vitamin D; *, *p* < 0.05; **, *p* < 0.01; ***, *p* < 0.001.

**Figure 3:**
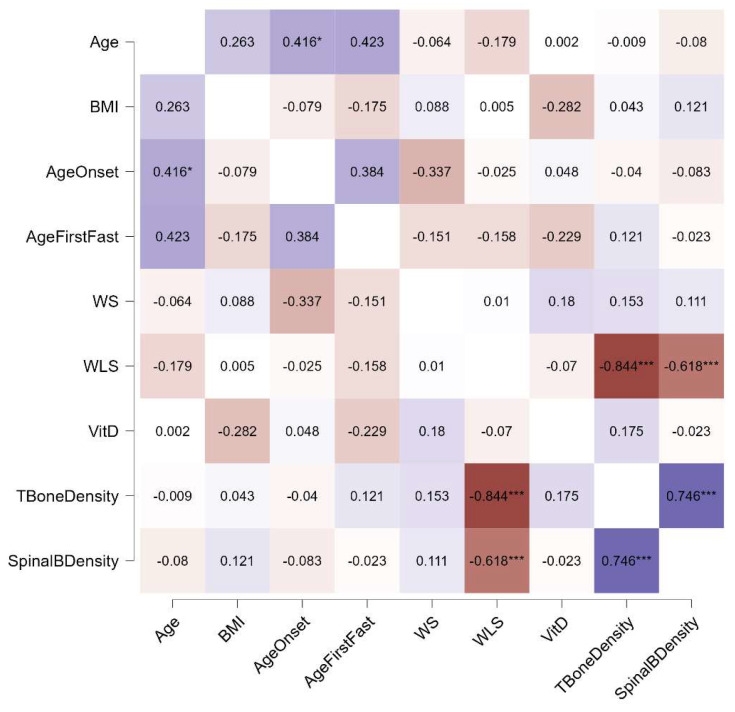
Spearman’s rho correlations for participants with BED. Purple indicates positive correlations, red indicates negative correlations, and the intensity of the colors reflects the strength of the correlation. WS, weight suppression; WLS, weight loss speed; VitD, vitamin D; *, *p* < 0.05; ***, *p* < 0.001.

Distinct patterns emerged in the relationships between bone density and other variables across the three diagnostic groups. In individuals with AN, both weight suppression and weight loss speed were significantly associated with total and spinal bone density, whereas in those with BN and BED, only weight loss speed showed such associations. Additionally, a link between current BMI and bone density was observed exclusively in the AN group. Notably, the age of onset was connected to the age of the first fasting episode in individuals with AN and BN, but not in those with BED. No correlations with current vitamin D levels were found in any group. These relationships, stratified by diagnosis, are illustrated in Figure 1, Figure 2 and Figure 3.

### 3.3. Regression Analyses

For each diagnosis, separate regression analyses were conducted for total bone density and spinal bone density, with WS and WLS as independent variables. In individuals with AN, the regression model for total bone density was significant (F = 39.02, *p* < 0.001, R^2^ = 0.36), with both WS (B = −0.19, *p* = 0.050) and WLS (B = −0.23, *p* < 0.001) identified as significant predictors. The model was significant for individuals with BN (F = 8.36, *p* = 0.001, R^2^ = 0.263), with WLS predictor of total bone density (B = −0.26, *p* < 0.001), while WS was not significant (B = 0.01, *p* = 0.508). Also, for BED the model was significant (F = 26.15, *p* < 0.001, R^2^ = 0.695), with WLS significant predictor (B = −0.49, *p* < 0.001), while WS was not significant (B = −0.01, *p* = 0.678). A similar pattern was observed for spinal bone density. In individuals with AN, the regression model was significant (F = 14.96, *p* < 0.001, R^2^ = 0.177), with WS (B = −0.27, *p* = 0.049) and WLS as predictors (B = −0.119, *p* < 0.001). For individuals with BN the model was also significant (F = 3.64, *p* = 0.033, R^2^ = 0.119), with WLS predictor (B = −0.19, *p* = 0.009), while WS was not significant (B = 0.01, *p* = 0.908). The same was for BED (F = 9.56, *p* = 0.001, R^2^ = 0.454), with WLS as predictor (B = −0.47, *p* < 0.001), while WS was not significant (B = 0.01, *p* = 0.993).

We conducted separate regression analyses for the Z-scores of the total and spinal bone density, incorporating additional collected features. The models included age, BMI, age of onset, maximum weight, and minimum weight. Among individuals with AN, significant regression models were found for both total bone density (F = 7.52, *p* < 0.001, R^2^ = 0.200) and spinal bone density (F = 6.17, *p* < 0.001, R^2^ = 0.143). Minimum weight emerged as a significant predictor for both total bone density (B = 0.06, *p* = 0.013) and spinal bone density (B = 0.05, *p* = 0.049). Similarly, age was a significant predictor for both outcomes (total bone density: B = −0.03, *p* = 0.026; spinal bone density: B = −0.02, *p* = 0.034). Maximum weight predicted total bone density exclusively (B = 0.02, *p* = 0.035), while current BMI was a significant predictor only for spinal bone density (B = 0.122, *p* = 0.045). In individuals with BN, both models were significant (total bone density F = 4.12, *p* = 0.004, R^2^ = 0.253; spinal bone density F = 5.01, *p* = 0.001, R^2^ = 0.303), with minimum weight emerging as the sole significant predictor for total bone density (B = 0.08, *p* = 0.003) and spinal bone density (B = 0.10, *p* < 0.001). In individuals with BED, no significant regression models were found for either total or spinal bone density.

## 4. Discussion

This study investigated the relationships between WS, WLS, and bone density in individuals with EDs. The findings reveal diagnostic-specific patterns that underscore the complex interplay between weight-related factors and bone health in ED populations.

Our results indicate that in individuals with AN, both WS and WLS are critical predictors of reduced bone density. Higher WS and faster WLS were associated with lower total and spinal bone density, consistent with prior research emphasizing the detrimental effects of prolonged low weight and rapid weight changes on bone metabolism [13]. Notably, the literature suggests that the speed of weight suppression itself may pose a significant risk factor for bone fragility [33,34,35]. Rapid reductions in body weight may exacerbate bone resorption, disrupt bone remodeling processes, and impair bone formation due to acute hormonal and metabolic imbalances [22,36]. These findings highlight the vulnerability of individuals with AN to severe bone health complications, driven by chronic energy deficits, nutritional deficiencies, and hormonal disruptions.

In contrast, among individuals with BN and BED, only WLS was significantly associated with bone density. Regression analyses revealed that in BN, minimum weight was the sole significant predictor of both total and spinal bone density, while no significant predictors or models were identified for BED. This suggests that while the rate of weight loss plays a role in bone health across EDs, the mechanisms underlying bone density changes differ by diagnosis. In AN, chronic malnutrition and sustained low weight are central to bone loss. In BN and BED, the transient nature of weight fluctuations, the lower WS and WLS, and differing hormonal profiles may mitigate some of the impacts on bone density [22,37]. Additionally, individuals with BED may be protected against bone loss due to their higher BMI, though this hypothesis warrants further investigation, and recent literature suggests that it may be overly simplistic [38].

Interestingly, regression analyses for AN identified minimum weight as a significant predictor of both total and spinal bone density, alongside WS and WLS. This highlights the critical importance of achieving and maintaining a healthy minimum weight for preserving bone health in individuals with AN, and the long-term risk connected to a low BMI [39,40]. The lack of significant relationships between vitamin D levels and bone density across all groups is notable and may be explained by variability in supplementation, sun exposure, or seasonal factors that were not controlled for in this study [41].

The observed differences between the total and spinal bone density outcomes may reflect the distinct vulnerabilities of weight-bearing versus non-weight-bearing bones. The literature suggests that spinal bone density, which primarily consists of trabecular bone, is particularly sensitive to hormonal disturbances such as estrogen deficiency and hypercortisolemia, both of which are common in AN [42,43,44]. These hormonal imbalances may disproportionately affect spinal bone density, making it more susceptible to the effects of WS and WLS compared to weight-bearing bones [45].

### 4.1. Clinical Implications

The findings have several important implications for the management of EDs. First, they highlight the need for clinicians to monitor WS and WLS across ED populations, particularly in individuals with AN, where these factors were strongly associated with reduced bone density. Stabilizing weight and minimizing rapid weight loss should be prioritized to mitigate bone fragility and reduce long-term health risks.

Second, the identification of the minimum weight as a critical predictor of bone density highlights the importance of avoiding extremely low body weight in the treatment of anorexia nervosa. Maintaining a weight that is not excessively low is crucial for improving bone health outcomes in this vulnerable population.

Third, routine DXA scans for assessing bone density should be considered in individuals with ED, especially those with high WS or rapid WLS. Early detection of bone health issues could enable timely interventions and reduce the risk of fractures and other complications.

Fourth, recent advances in medical management, such as accelerated refeeding protocols, have shown promise in improving weight restoration without increasing the risk of refeeding syndrome [46]. Additionally, the use of olanzapine has been found to facilitate weight restoration more effectively than placebo, without the adverse metabolic effects observed in individuals without starvation [47]. These approaches highlight the potential for integrating pharmacological and nutritional strategies to address the medical complications associated with EDs, including bone health [48].

Finally, given the associations between WLS and bone density observed in BN and BED, clinicians should remain vigilant for potential bone health issues in these groups, even though the underlying mechanisms may differ. Larger longitudinal studies are needed to refine prevention strategies for these populations.

### 4.2. Limitations

Several limitations of this study should be noted. The cross-sectional design precludes conclusions about causality, and longitudinal studies are needed to clarify the temporal relationships between WS, WLS, and bone density. Additionally, the relatively small sample sizes for BN and BED may limit the generalizability of findings to these groups.

## 5. Conclusions

This study highlights the significant impact of WS and WLS on bone density in individuals with AN, underscoring the need for targeted interventions to address bone health in this population. By identifying weight-related factors as key contributors to bone density loss, this research provides a foundation for developing integrated treatment approaches that address both the medical and psychological needs of individuals with EDs. Protecting bone health is essential not only for improving quality of life but also for supporting long-term recovery and reducing the risk of lifelong complications associated with EDs.

Future research should explore the role of hormonal and metabolic mediators, such as estrogen and leptin, in the observed relationships and investigate potential interventions to protect bone health in individuals with EDs. Moreover, studies should examine the longitudinal effects of weight restoration and stabilization on bone density outcomes, as well as the interplay between nutritional interventions, physical activity levels, and bone health across different ED populations. These investigations may provide deeper insights into effective prevention and treatment strategies for this vulnerable population.

## Figures and Tables

**Table 1:** Description of the sample.

	AN n = 187	BN n = 57	BED n = 26	H *p*	η^2^	Post Hoc
Age, years	26.25 10.22	25.97 10.94	39.23 15.15	18.41 <0.001	0.112	AN < BED (<0.001) BN < BED (<0.001)
BMI, kg/m^2^	15.21 1.86	21.02 1.95	35.48 8.33	176.87 <0.001	0.792	AN < BN (<0.001) AN < BED (<0.001)
Age of onset, years	17.53 5.97	16.55 5.96	21.00 14.25	2.39 0.303	0.025	
Maximum weight, kg	58.03 10.68	68.88 13.23	107.29 29.30	73.49 <0.001	0.526	AN < BN (<0.001) AN < BED (<0.001) BN < BED (0.002)
Minimum weight, kg	37.23 5.58	45.35 7.39	62.41 15.70	83.78 <0.001	0.514	AN < BN (<0.001) AN < BED (<0.001) BN < BED (0.024)
Age of first fasting	17.44 6.86	17.02 7.66	17.94 6.64	1.05 0.593	0.001	
Weight suppression (WS), kg	15.17 8.14	18.25 10.83	9.85 3.53	15.31 <0.001	0.526	BED < AN (0.005) BED < BN (< 0.001)
Weight loss speed (WLS), kg/months	4.28 3.75	3.63 2.30	2.61 1.67	5.486 0.048	0.514	
Total Body Bone Density	−0.59 1.40	−0.06 1.24	0.25 0.98	14.63 0.001	0.048	AN < BN (0.028) AN < BED (0.004)
Spinal Bone Density	−1.49 1.27	−0.53 1.25	0.50 1.170	53.78 <0.001	0.210	AN < BN (<0.001) AN < BED (<0.001) BN < BED (0.013)
Vit. D, ng/mL	27.86 14.66	25.02 10.41	23.43 18.34	7.12 0.028	0.013	BED < AN (0.024)

AN, anorexia nervosa; BN, bulimia nervosa; BED, binge eating disorder.

## Data Availability

Data supporting the findings of this study are available from the corresponding author, upon reasonable request.
